# Hospitalization with infections and risk of Dementia: a systematic review and meta-analysis

**DOI:** 10.18632/aging.206329

**Published:** 2025-10-13

**Authors:** Wei Yu Chua, Jia Dong James Wang, Claire Kar Min Chan, Ling-Ling Chan, Eng-King Tan

**Affiliations:** 1 Yong Loo Lin School of Medicine, National University of Singapore, Singapore, Singapore; 2 Lee Kong Chian School of Medicine, Nanyang Technological University, Singapore, Singapore; 3 Department of Diagnostic Radiology, Singapore General Hospital Campus, National Neuroscience Institute, Singapore, Singapore; 4 Department of Neurology, Singapore General Hospital Campus, National Neuroscience Institute, Singapore, Singapore; 5 Neuroscience and Behavioral Disorders, Duke-NUS Medical School, Singapore, Singapore

**Keywords:** Alzheimer disease, Dementia, hospitalization, infections, meta-analysis

## Abstract

Background: Dementia affects more than 50 million people worldwide, with 10 million new diagnosis each year. The link between hospitalization with infections and risk of Dementia is unclear. We conducted a meta-analysis on the association between hospitalization with infection and risk of Dementia.

Methods: We searched MEDLINE and Embase from inception to March 31, 2025 to identify cohort studies comparing the frequency of Dementia in patients hospitalized with infections with those without. We computed hazard ratios (HR) for each study and pooled the results using a random-effects meta-analysis.

Results: Out of 1900 studies that were screened initially, 16 studies comprising 4,266,276 patients were included for analysis. Hospitalization with infection was associated with an increased risk of all-cause Dementia (HR: 1.83, 95% CI: 1.58–2.13, *p* < 0.0001), Alzheimer’s Disease (AD) (HR: 1.60, 95% CI: 1.23–2.08, *p* < 0.001) and Vascular Dementia (HR: 3.68, 95% CI: 2.16–6.27, *p* < 0.001). Among the infections, having Sepsis (HR: 1.78, 95% CI: 1.53–2.08) was associated with the highest risk of all-cause Dementia, followed by Pneumonia, Urinary Tract Infections, Skin and Soft Tissue infections.

Conclusions: Our meta-analysis showed that hospitalization with infection was associated with increased risk of Dementia. Sepsis carried the highest risk, followed by Pneumonia, Urinary Tract Infections, Skin and Soft Tissue infections.

## INTRODUCTION

According to the World Health Organization (WHO), Dementia affects over 50 million individuals globally, with Alzheimer’s Disease (AD) being the most prevalent subtype [1]. In adults above 65 years old, Dementia remains the fifth leading cause of death in America, with estimated total healthcare costs exceeding USD $300 billion annually [2]. The cost of Dementia is expected to exceed USD $1 trillion, with significant implications for healthcare systems and caregivers as the global population ages [3]. Furthermore, as aging occurs, infections become more frequent and severe [4]. These infections accelerate aging through mechanisms that include enhanced inflammation, pathogen-dependent tissue destruction, and accelerated cellular ageing through increased turnover [4].

Developed countries such as the United States of America and Singapore have rapidly aging populations. While adults above 65 years old account for less than 20% of this population, they account for more than 40% of hospitalizations [5, 6]. In addition, hospitalization for infection is an increasingly common cause of emergency admission, morbidity and mortality in these cohorts [7].

With rising admission rates among adults above 65 years old for infection-related conditions and the increasing prevalence of Dementia, the relationship between hospitalization for infection and risk of Dementia has been increasingly studied. Since the Covid-19 pandemic, the link between hospitalization with infections and risk of Dementia has attracted considerable attention. A recent meta-analysis by Zhang et al. found a significant link between Covid-19 infection and new-onset Dementia, especially in individuals older than 65 years [8]. While this highlights the need for cognitive monitoring and early intervention for Covid-19 survivors to address potential long-term neurological impacts, the evidence for other non-Covid-19 related infections remains unaddressed. Previous analyses on hospitalization with infection and risk of Dementia lacked large-scale longitudinal studies and included studies with poor quality of evidence [9] and did not examine the subtypes of infection with the highest risk of Dementia. With the publication of several large-scale longitudinal studies in recent years [10–12], we can perform the first meta-analysis on subtype of infections on risk of all-cause Dementia as well as the impact of hospitalization on different subtypes of Dementia.

Here we conducted the first meta-analysis which included large-scale longitudinal studies in the past 5 years, to determine the following outcomes: (1) the impact of hospitalization with infections and risk of Dementia (2) and relationship between subtypes of infections and risk of Dementia.

## MATERIALS AND METHODS

We registered this meta-analysis with PROSPERO at CRD420251022942 and followed the reporting guidelines of Preferred Reporting Items for Systematic Reviews and Meta-analyses (PRISMA) [13].

### Information source and search strategy

A systematic search was conducted in MEDLINE and Embase using keywords and MeSH terms synonymous with “Hospitalization”, “Infection” and “Dementia”, which formed the basis of the search strategy. The search period includes articles from inception to March 31, 2025. Only full-text articles published in English were included. The complete search strategy and search terms can be found in Supplementary Table 1. References were imported into EndNoteX9 and Covidence for the initial removal of duplicates [14].

### Study selection

Two authors (WYC and JDJW) reviewed each reference in a blinded manner, and disagreements were resolved through discussion or referred to a third independent author for the final decision (CKMC). Study selection occurred in two phases: initial screening of titles and abstracts, followed by full-text review of potentially eligible studies. Accepted study designs included case-control and cohort studies. Original studies in English discussing hospitalization with infections in adults were included. Studies on all-cause Dementia, AD and Vascular Dementia were included. We excluded studies on Covid-19, non-peer-reviewed articles, review articles, and conference abstracts. Studies on patients younger than 18 years, including patients with known Dementia or lacked data about the risk of Dementia, were excluded. Studies involving non-human or animal were also excluded.

### Data extraction

Data extraction was conducted independently by two investigators (WYC and JDJW). The data collected included sample size, year of publication, total number of participants, age and sex of study participants, reason for hospitalization, and risk of Dementia. If multivariate analysis was utilized, the hazard ratio selected would be from the model with the most variables included. A third author (CKMC) was consulted to make the final decision regarding any discrepancies.

### Quality assessment

The Joanna Briggs Institute Critical Appraisal Tools were used for quality assessment [14, 15]. Two investigators (CKMC and JDJW) independently reviewed all included studies and a third independent author (WYC) was consulted regarding any discrepancies. The maximum score attainable (signifying high quality) is 8 points for analytical cross-sectional studies, 10 points for case–control studies, and 11 points for cohort studies.

### Data analysis

All analyses were undertaken using R software version 4.4.3. The statistical packages used in R were (tidyverse; meta; metafor; ggplot2; gridExtra and dmetar) [16]. To estimate the pooled risk ratios and its corresponding 95% confidence intervals (CIs), we used the random effects model where applicable. A hazard ratio >1 indicates that hospitalization with infection is associated with a higher risk of Dementia. The proportion of variability due to heterogeneity was assessed using I^2^ and we considered heterogeneity to be significant when the I^2^ statistic was ≥25%. Precalculated log-transformed hazards ratios (HRs) were pooled using the inverse variance method [16]. The level of significance is defined as *p* < 0.05. All results were presented as their effect sizes with the accompanying 95% CIs.

For meta-analyses with high heterogeneity, we conducted influence analysis to determine the contribution of each study to overall heterogeneity. From the Baujat plots, leave-one-out analyses, and inspection of the forest plots, we performed a sensitivity analysis in which outliers were excluded [16]. If there are studies from overlapping databases, we only included the study with the largest sample size for the respective analysis.

### Data availability

The datasets used and/or analysed during the current study are available from the corresponding author on reasonable request.

## RESULTS

### Overview

In total, 1900 studies were found after searching MEDLINE and Embase. Of which, 744 were duplicates, and 1156 studies remained following duplicate removal. The study team screened the titles and abstracts of these studies and included 30 studies for further review. The study team retrieved the full texts of these 30 studies and 16 studies [10–12, 17–29] involving 4,266,276 patients (1,200,112 patients hospitalized with infection and 3,066,154 controls) were included in the final analysis (Supplementary Figure 1).

### Characteristics of included studies

A summary of the characteristics of included studies can be found in Table 1. Of the 16 studies, 6 were conducted in the United Kingdom, 5 were from United States of America, 2 were from Taiwan and the remaining 3 were from Finland, Netherlands and New Zealand. With the exception of Chou et al. and Chalitsios et al. [18, 19], all the studies were cohort studies. 10 studies were prospective studies while the remaining 6 were retrospective studies. Majority of the studies focused on any infection with only Richmond-Rakerd et al., Sipila et al. and Wang et al. reported separate data on viral and bacterial infections [10, 26, 29]. Of the 16 studies, studies frequently reported on all-cause Dementia while only 6 and 3 studies reported on AD [10, 17, 19, 23, 26, 29] and Vascular Dementia [10, 26, 29] respectively. The mean age of the patients hospitalized with infections in majority of the studies are in the ranges of 60–75 years old. The median duration of follow-up varied among studies, ranging from 1.7 years to 25 years. The largest study included 1,742,406 participants [26] while the smallest study included 331 participants [25]. Both Beydoun et al. and Wang et al. extracted data from the UK Biobank database, with Beydoun et al. having the larger database [17, 29]. Similarly, Chalitsios et al., Morton et al. and Muzambi et al. utilized the Clinical Practice Research Datalink database, with Muzambi et al. having the largest database. A summary of the quality of studies using the Joanna Briggs Institute (JBI) Critical Appraisal Tools can be found in Table 1.

**Table 1:** Summary of included studies.

**Study**	**Country**	**Study design**	**Study type**	**Study period**	**Study setting**	**Total sample size, No.**	**Female, No. (%)**	Age, y*	**Median duration of follow up, y^†^**	**Hospitalized with infection, No.**	**Controls, No.**	**JBI scoring^§^**
Beydoun et al., 2023	United Kingdom	Prospective	Cohort	2006 to 2021	UK Biobank	355046	190,124 (53.5)	60.4 ± 0.01	>3 years	42605	312441	11/11
Bohn et al., 2023	United States of America	Prospective	Cohort	1987 to 2019	Atherosclerosis Risk in Communities (ARIC) study	15688	8658 (55.2)	54.7 ± 5.8	25.1 (22.2–29.1)	5999	9689	11/11
Chalitsios et al., 2023	United Kingdom	Retrospective	Case-Control	2002 to 2017	Clinical Practice Research Datalink and Hospital Episode Statistics	261976	132639 (50.6)	69.8 ± 17.8	1.7 (0.5–4)	55808	206168	10/10
Chou et al., 2017	Taiwan	Retrospective	Case-Control	2001 to 2011	Longitudinal Health Insurance Database (LHID)	61398	26997 (44.0)	≥65	10	20466	40932	9/10
Guerra et al., 2012	United States of America	Retrospective	Cohort	2005 to 2008	Medicare Standard Analytic Files (SAF) from the Centers for Medicare and Medicaid Services (CMS).	25368	13275 (52.3)	76.6 ± 6.8	2.5 ± 0.9	8217	17,151	10/11
Mawanda et al., 2016	United States of America	Retrospective	Cohort	2002 to 2004	Veterans Health Administration national repository	417172	6636 (2.1)	67.7 ± 8.1	9.03 ± 1.1	87400	329,772	10/11
Morton et al., 2023	United Kingdom	Retrospective	Cohort	2005 to 2016	Clinical Practice Research Datalink and Hospital Episode Statistics	49916	35075 (70.3)	72.6 ± 12.7	2.60 (0.97–4.75)	10493	39,423	11/11
Muzambi et al., 2021	United Kingdom	Retrospective	Cohort	2004 to 2018	Clinical Practice Research Datalink and Hospital Episode Statistics	989800	537602 (54.3)	71.7 ± 7.9	5.2 (2.3–9.0)	402204	587596	11/11
Ou et al., 2021	Taiwan	Retrospective	Cohort	2000 to 2015	National Health Insurance Research Database (NHIRD)	6628	2620 (39.5)	65.67	15	1657	4971	10/11
Pendlebury et al., 2024	United Kingdom	Prospective	Cohort	2002 to 2012	Oxford Vascular Study	1369	674 (49.2)	72 ± 13	4.87	236	1133	10/11
Peters et al., 2022	Netherlands	Prospective	Cohort	2006 to 2015	Radboud University Nijmegen Diffusion Tensor and Magnetic Resonance Cohort study	331	142 (43)	64.2 ± 8.3	9	270	61	10/11
Richmond-Rakerd et al., 2024	New Zealand	Prospective	Cohort	1989 to 2019	New Zealand-based population register study	1,742,406	860529 (49.4)	61–70^‡^	30	465330	1,277,076	11/11
Shah et al., 2013	United States of America	Prospective	Cohort	1997 to 2008	Cardiovascular Health Study	5888	3393 (57.6)	72.8 ± 5.6	10	639	5249	10/11
Sipila et al., 2021	Finland	Prospective	Cohort	1986 to 2005	Finnish Public Sector study, Health and Social Support study, Still Working study	260490	182976 (70.2)	18–39^‡^	15.4	77108	183382	11/11
Tate et al., 2014	United States of America	Prospective	Cohort	2000 to 2008	Gingko Effect on Memory study	3069	1418 (46.2)	78.5	6.1	221	2848	10/11
Wang et al., 2025	United Kingdom	Prospective	Cohort	2006 to 2019	UK Biobank	69,731	34752 (49.8)	59.59 ± 7.07	10.75	21469	48262	11/11

^*^Mean ± SE. ^†^Mean ± SD/Median, (IQR); ^‡^50th percentile Range. ^§^JBI (Joanna Briggs Institute). Abbreviation: NA: Not applicable.

### Definition of exposure

All studies included in the analysis provided data on hospitalization with infections. The International Classification of Diseases (ICD) 9th and 10th Edition revision codes were commonly used to identify diagnosis of infections from hospital records. A detailed definition of the hospitalization with infection for each study can be found in Supplementary Table 2.

### Definition of control

All studies included in the analysis compared hospitalization with infections against a control group. A detailed definition of the controls in each study can be found in Supplementary Table 2.

### Diagnosis of Dementia

The ICD 9th and 10th Edition revision codes were commonly used to identify diagnosis of Dementia, AD and Vascular Dementia from hospital records. All studies excluded patients with Dementia at baseline. Detailed exclusion criteria for each study can be found in Supplementary Table 2.

### Primary outcome

**Figure 1:**
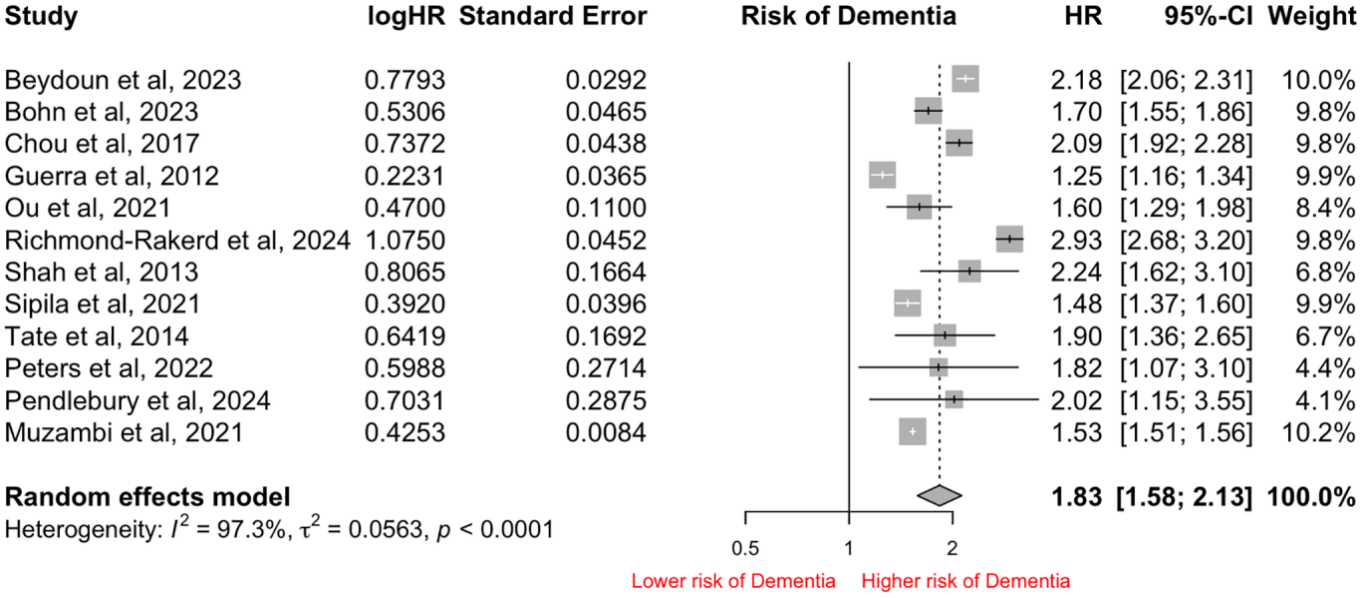
Forest Plot of pooled Hazard Ratio (HR) for risk of all-cause Dementia in patients hospitalized with infections after sensitivity analysis and excluding overlapping databases.

16 studies involving 4,266,276 patients were pooled and we found that patients hospitalized with infections were at higher risk of developing all-cause Dementia (HR: 1.83, 95% CI: 1.56–2.14, *p* < 0.001). The I^2^ index was 98.4% and the Cochran *Q*-test was significant at *p* < 0.0001 (Supplementary Figure 2). Influence analysis revealed 1 outlier, Mawanda et al. [21] and sensitivity analysis excluding the outlier was conducted (Supplementary Figures 3 and 4). After excluding Mawanda et al. and 3 studies with overlapping databases (Chalitsios et al. [18], Morton et al. [22] and Wang et al. [29]), results from 12 studies involving 3,467,481 patients were analyzed. We found that patients hospitalized with infections were at higher risk of developing all-cause Dementia (HR: 1.83, 95% CI: 1.58–2.13, *p* < 0.0001). The I^2^ index was 97.3% and the Cochran *Q*-test was significant at *p* < 0.0001 (Figure 1).

### Secondary outcomes: type of infection

**Figure 2:**
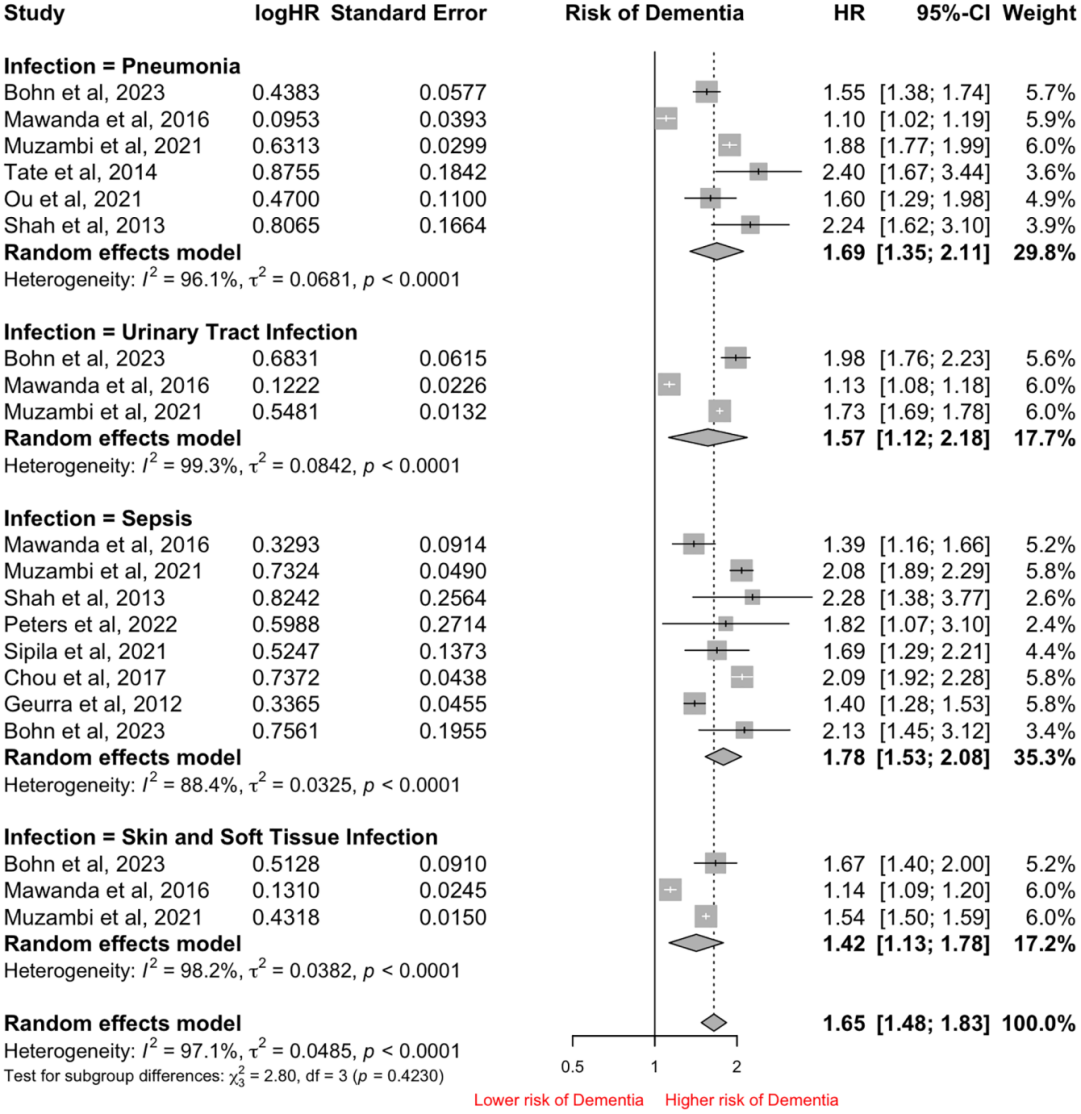
Forest Plot of pooled Hazard Ratio (HR) for risk of all-cause Dementia in patients hospitalized with infections based on subtype of infections.

Excluding the 3 studies with overlapping databases, subgroup analyses were conducted in the remaining 13 studies. Sepsis (*n* = 8) was the most frequently reported outcome, followed by Pneumonia (*n* = 6), Urinary Tract Infection (*n* = 3), and Skin and Soft Tissue infections (*n* = 3). Among the infections, having Sepsis (HR: 1.78, 95% CI: 1.53–2.08) was associated with the highest risk of all-cause Dementia, followed by Pneumonia (HR: 1.69, 95% CI: 1.35–2.11), Urinary Tract Infection (HR: 1.57, 95% CI: 1.12–2.18) and Skin and Soft Tissue infections (HR: 1.42, 95% CI: 1.13–1.78). The I^2^ index was 97.1% and the Cochran *Q*-test was significant at *p* < 0.01 (Figure 2).

Of the 8 studies reporting sepsis outcomes, Shah et al. and Guerra et al. reported on severe sepsis and the risk of developing all-cause Dementia. There was no significant difference seen in patients hospitalized with sepsis or severe sepsis (HR: 1.85, 95% CI: 1.58–2.17 vs. HR: 1.06, 95% CI: 1.06–2.65, *p* = 0.68) and the risk of developing all-cause Dementia (Supplementary Figure 5).

There are only 3 and 4 studies respectively comparing the risk of only viral [10, 26, 29] or bacterial infections [10, 23, 26, 29] in developing all-cause Dementia. There were no significant differences in the impact of hospitalization with either viral or bacterial infection (HR: 3.08, 95% CI: 1.64–5.81 vs. HR: 2.35, 95% CI: 1.47–3.74, *p* = 0.50) in developing all-cause Dementia (Supplementary Figure 6).

### Subgroup analysis: subtype of Dementia

**Figure 3:**
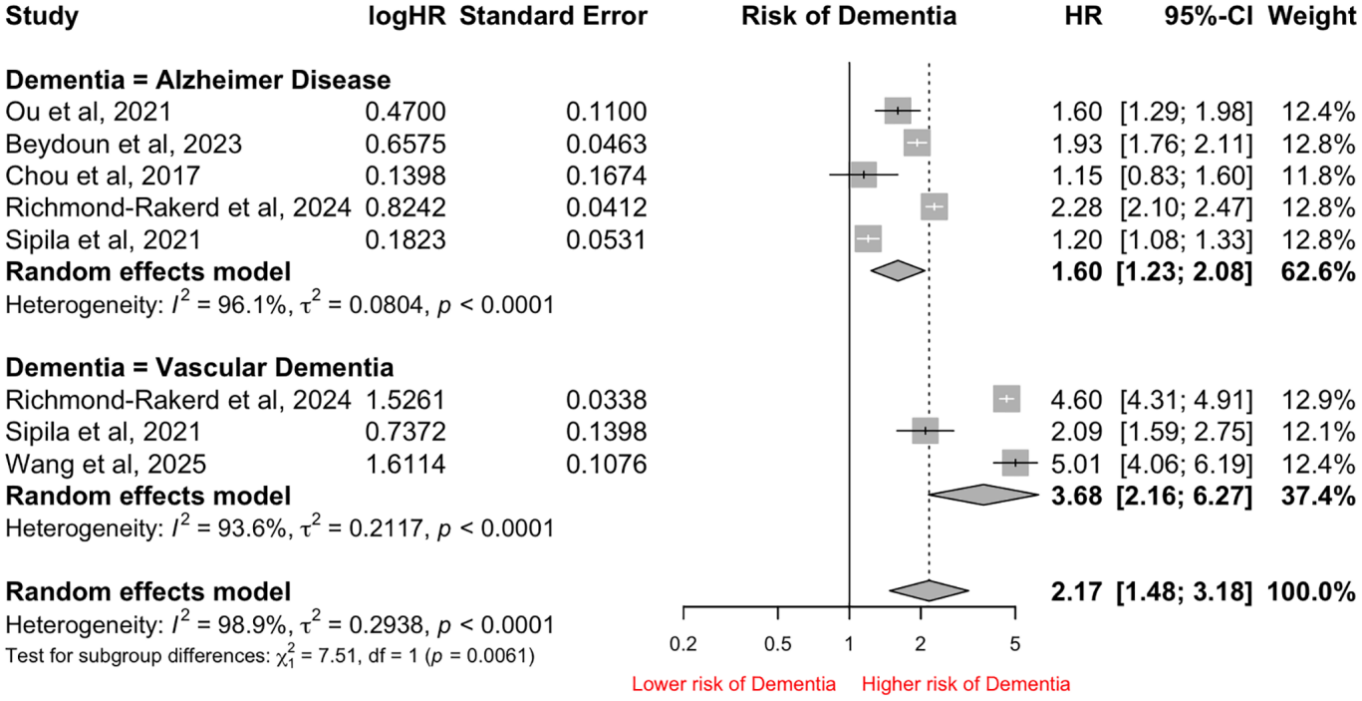
Forest Plot of pooled Hazard Ratio (HR) for risk of different subtypes of Dementia in patients hospitalized with infections.

While majority of the studies reported on all-cause Dementia, 6 and 3 studies reported on AD [10, 17, 19, 23, 26, 29] and Vascular Dementia [10, 26, 29] respectively. As both Beydoun et al. and Wang et al. share the same database, Wang et al. was excluded from the analysis of AD. Our analysis found that patients hospitalized with infection were at significantly higher risk of developing both forms of Dementia. However, patients hospitalized with infection were at significantly higher risk of developing Vascular Dementia compared to AD (HR: 3.68, 95% CI: 2.16–6.27 vs. HR: 1.60, 95% CI: 1.23–2.08, *p* = 0.0061). The I^2^ index was 98.9% and the Cochran *Q*-test was significant at *p* < 0.0001 (Figure 3).

### Subgroup analysis: duration of follow-up

Of the 13 studies, 5 studies had median follow-up duration of 10 or more years [10, 12, 17, 26, 27] while the remaining 8 studies had a duration of less than 10 years [11, 19–21, 23–25, 28]. Studies with 10 or more years of follow-up had significantly higher risk of patients hospitalized with infections developing all-cause Dementia (HR: 2.04, 95% CI: 1.60–2.60 vs. HR: 1.57, 95% CI: 1.33–1.86, *p* < 0.0001) compared to studies with less than 10 years. The I^2^ index was 98.2% and the Cochran *Q*-test was significant at *p* < 0.0001 (Supplementary Figure 7).

### Subgroup analysis: study design

Majority of the 13 studies were conducted prospectively (*n* = 8). Compared to Retrospective studies, Prospective studies had significantly higher risk of patients hospitalized with infections developing all-cause Dementia (HR: 2.00, 95% CI: 1.67–2.40 vs. HR: 1.49, 95% CI: 1.22–1.83, *p* < 0.01). The I^2^ index was 98.2% and the Cochran *Q*-test was significant at *p* < 0.0001 (Supplementary Figure 8).

### Subgroup analysis: study size

Due to differing sample sizes, we conducted a subgroup analysis based on the sample sizes (≥10,000 and <10,000). We found no significant differences between studies of different sample sizes in the risk of developing all-cause Dementia (HR: 1.70, 95% CI: 1.41–2.06 vs. HR: 2.02, 95% CI: 1.66–2.47, *p* = 0.22). The I^2^ index was 98.2% and the Cochran *Q*-test was significant at *p* < 0.0001 (Supplementary Figure 9).

### Subgroup analysis: region

4 studies were conducted in Europe, while 6 studies were conducted in North America and the remaining 3 studies were conducted in the Asia-Pacific region. Based on our meta-analysis, there were no significant difference in the risk of patients hospitalized with infection developing all-cause Dementia among the 3 regions (HR: 1.53, 95% CI: 1.50–1.55 vs. HR: 1.66, 95% CI: 1.32–2.08, vs. HR: 2.16, 95% CI: 1.54–3.03, *p* = 0.10). The I^2^ index was 98.2% and the Cochran *Q*-test was significant at *p* < 0.0011 (Supplementary Figure 10).

## DISCUSSION

To investigate the association between the hospitalization with infection and risk of Dementia, we conducted a systematic review and meta-analysis involving 16 studies and 4,266,276 patients to determine if hospitalization with infection is associated with increased risk of developing Dementia. We found that hospitalization with infection was associated with increased risk of all-cause Dementia (HR: 1.83, 95% CI: 1.58–2.13, *p* < 0.0001), AD (HR: 1.60, 95% CI: 1.23–2.08, *p* < 0.001) and Vascular Dementia (HR: 3.68, 95% CI: 2.16–6.27, *p* < 0.001) compared to patients that were not hospitalized with infections. Among the infections, having Sepsis (HR: 1.78, 95% CI: 1.53–2.08) was associated with the highest risk of developing all-cause Dementia, followed by Pneumonia (HR: 1.69, 95% CI: 1.35–2.11), Urinary Tract Infections (HR: 1.57, 95% CI: 1.12–2.18) and Skin and Soft Tissue infections (HR: 1.42, 95% CI: 1.13–1.78).

Our meta-analysis is the first to provide a comprehensive analysis on the impact of hospitalization with infection and risk of developing all-cause Dementia, AD and Vascular Dementia. Our study is also the first to provide HRs on the impact of various common infections on the risk of developing all-cause Dementia. Infections in the elderly tend to result in greater morbidity and mortality, accounting for one third of all deaths in people 65 years and older [30]. The impact of an infection on elderly can result in functional decline, increased risk of myocardial infarction, stroke, and exacerbate existing chronic conditions over time [31]. Based on multiple large scale longitudinal studies conducted in Europe and Asia-Pacific [10, 26, 29], there is increasing evidence of the impact of hospitalization with infection on the risk of all-cause Dementia. In the elderly population, infections involving the urinary tract, respiratory system, skin and soft tissue infections are the most common reason for hospitalization [31]. Based on this, our meta-analysis found that among the common infections, Sepsis (HR: 1.78, 95% CI: 1.53–2.08) was associated with the highest risk of developing all-cause Dementia, followed by Pneumonia (HR: 1.69, 95% CI: 1.35–2.11), Urinary Tract Infections (HR: 1.57, 95% CI: 1.12–2.18), Skin and Soft Tissue infections (HR: 1.42, 95% CI: 1.13–1.78) indicating that all infections are associated with risk of all-cause Dementia. While severity of infection may be a contributing factor to all-cause Dementia, our study did not find any significant difference in patients hospitalized with sepsis or severe sepsis.

Two studies from the Clinical Practice Research Datalink database, Muzambi et al. and Morton et al., compared the frequency of hospitalization for infection and risk of all-cause Dementia. Muzambi et al. found that the incident infection carries the highest risk (HR: 1.34, 95% CI: 1.32–1.37) while subsequent infection carried a lower risk (HR: 1.02, 95% CI: 1.01–1.02) of all-cause Dementia. However, in the study by Morton et al. which included only post-stroke patients, the incident infection carried the lowest risk (HR: 1.27, 95% CI: 1.13–1.43) while subsequent 2nd, 3rd and 4th infections carried a higher HR of 1.31, 1.50 and 1.71 respectively. Although the number of studies is limited, the differing impacts of subsequent infections observed may be explained by the possibility that a pre-existing stroke accelerates the progression to all-cause Dementia in patients experiencing repeated infections [32]. Therefore, more research on the frequency of hospitalization for infection and risk of all-cause Dementia in different subgroups should be studied.

The relationship between systemic inflammation and neurodegeneration is well supported by our meta-analysis. Our study found that hospitalization with infection was associated with increased risk of all-cause Dementia (HR: 1.69, 95% CI: 1.35–2.11, *p* < 0.0001), AD (HR: 1.60, 95% CI: 1.23–2.08, *p* < 0.001) and Vascular Dementia (HR: 3.68, 95% CI: 2.16–6.27, *p* < 0.001). In recent years, there is growing body of evidence that shows the infiltration of pathogens may trigger AD. Firstly, pathogens may directly weaken the blood–brain barrier, and cross into the central nervous system, causing neurological damage by stimulating neuroinflammation. Secondly, pathogens may cross the intestinal barrier, reach systemic circulation, and cross into the blood-brain barrier. These pathogens may cause low-grade chronic inflammation and subsequently neuroinflammation from the periphery [33]. Particularly in AD, the most common form of Dementia, bridging of the blood-brain barrier by pathogens such as viruses, bacteria, fungi and parasites may increase the risk of symptomatic infections. It is possible that DNA/RNA, proteins, enzymes or lipopolysaccharides linked directly or indirectly to the pathogens may contribute to chronic low-grade neuroinflammation, resulting in enhanced production of amyloid-β (Aβ) and aggregation of hyperphosphorylated tau protein, resulting in neurodegeneration and cognitive decline [34]. Although certain viruses such as Herpes simplex virus (types 1 and 2), Human Herpes Virus 6, Varicella zoster virus (VZV), Human immunodeficiency virus (HIV), Epstein–Barr virus (EBV), cytomegalovirus (CMV) and hepatitis C virus (HCV) have been linked with development of AD, these viruses do not account for common hospital treated infections and should be further researched [35]. While the link between hospitalization with infections and Vascular Dementia is unclear, the association between hospitalization with infections and cardiovascular dysfunction and complications have been well studied [31]. These complications in turn lead to cerebrovascular disease, brain injury and the resulting disruption of cognitive networks may culminate in Vascular Dementia [36].

Data from WHO have found age to be the strongest risk factor for the development of Dementia [1]. However, it is not an inevitable consequence of biological aging and Dementia does not exclusively impact older people as young onset Dementia accounts for up to 9% of cases worldwide [1]. The duration of follow-up helps to better ascertain the impact of aging on the development of Dementia. While age could be a potential confounder [37], we selected hazard ratios that adjusted for age in all of the studies included in our meta-analysis. We found that studies with 10 or more years of follow-up had significantly higher risk of patients hospitalized with infections developing all-cause Dementia (HR: 2.04, 95% CI: 1.60–2.60 vs. HR: 1.57, 95% CI: 1.33–1.86, *p* < 0.01) compared to studies with shorter follow-up duration (10 years or less). This highlights that even in shorter duration of time, the risk of all-cause Dementia increases in patients hospitalized with infections. Furthermore, Muzambi et al. found that the risk of all-cause Dementia is highest in the first year after infection (HR: 1.86, 95% CI: 1.80–1.92). The mean age of the patients hospitalized with infections in majority of the studies are in the ranges of 60–75 years old. A meta-analysis by Jorm et al. has shown that the incidence of all-cause Dementia and AD increases exponentially from 65 years old [37]. This indicates that a significant proportion of patients are already facing an exponential risk of developing Dementia at baseline, coupled with the fact that an exposure to hospitalization with infection significantly increases the risk of all-cause Dementia in the short term and even higher in the long term even after adjusting for age. This highlights the potential need to institute early Dementia prevention and treatment for at risk patients.

### Further directions

While our meta-analysis confirms that hospitalization with infections increases the risk of all-cause Dementia, we are unable to determine the impact of severity of infections. Our study found a significantly higher risk of hospitalization with infection with Vascular Dementia compared to AD. Further functional studies to identify novel pathophysiologic clues will be useful for diagnostic and therapeutic purposes. Clinical studies should also focus on early intervention and treatment for hospitalized elderly patients with infections to mitigate the risk of developing AD and Vascular Dementia [38, 39]. All the studies did not include other less common forms of Dementia such as Lewy Body Dementia or Frontotemporal Dementia. Further studies can consider analysing the impact of hospitalization with infections on these other subtypes of Dementia.

### Limitations

First, due to the characteristics of the studies included, we were unable to perform analysis based on severity of infections or duration of hospitalizations. Although severity of infections is an important factor to consider, majority of the studies did not distinguish between the severity of infections. While our study conducted subgroup analysis between the severity of sepsis, the limited number of studies requires careful interpretation of the analysis. Second, duration of follow-up varied among studies. Our analysis showed that studies of longer follow-up duration reported greater risk of developing all-cause Dementia compared to studies of shorter duration. We acknowledge the potential limitation of using median follow-up duration as a surrogate for follow-up duration. However, median follow-up duration is commonly reported across all studies. Third, while the majority of the studies reported on all-cause Dementia, only 6 and 3 studies separately reported AD [10, 17, 19, 23, 26, 29] and Vascular Dementia [10, 26, 29] respectively. Therefore, the pooled HR for the subgroup analysis on AD and Vascular Dementia may be heavily influenced by individual studies. Fourth, 5 studies in our meta-analysis had overlapping databases (Beydoun et al. and Wang et al. from the UK Biobank database while Chalitsios et al., Morton et al. and Muzambi et al. utilized the Clinical Practice Research Datalink database). To ensure that there is no overlapping data, we included the study with the largest sample size in the respective analysis. Fifth, due to the scope of this paper, we did not include non-hospitalization related infections such as Herpes Simplex Virus, Human Immunodeficiency Virus infections that have been shown to be associated with AD [40, 41]. Sixth, our study excluded Covid-19 related studies as this was the focus of a meta-analysis recently [8]. The meta-analysis found that Covid-19 infections increase the risk of developing new-onset Dementia (HR = 1.49, 95% CI: 1.33–1.68). Last, as our study included adjusted HR from all studies, the variables used in each model are different. To account for the possibility of heterogeneity between studies, our study conducted sensitivity analysis to account for the heterogeneity.

## CONCLUSIONS

Our meta-analysis involving 16 studies and 4,266,276 patients showed for the first time that hospitalization with infection increases the risk of all-cause Dementia, AD and Vascular Dementia. Among the infections, Sepsis carries the highest risk of developing all-cause Dementia, followed by Pneumonia, Urinary Tract Infections, Skin and Soft Tissue infections. Based on our findings, we suggest that early intervention and treatment for at risk hospitalized elderly patients with infections can potentially help to mitigate the risk of developing AD and Vascular Dementia.

## Supplementary Materials

Supplementary Figures

Supplementary Tables
