# Supplementary material for: Hospitalization with infections and risk of Dementia: a systematic review and meta-analysis
Source: Aging (Albany NY). 2025 Oct 13;17(10):2561–81. doi: 10.18632/aging.206329 (PMC12606967; doi:10.18632/aging.206329)
Supplement: Supplementary Figures [file aging-17-10-206329-s001.pdf]

SUPPLEMENTARY FIGURES

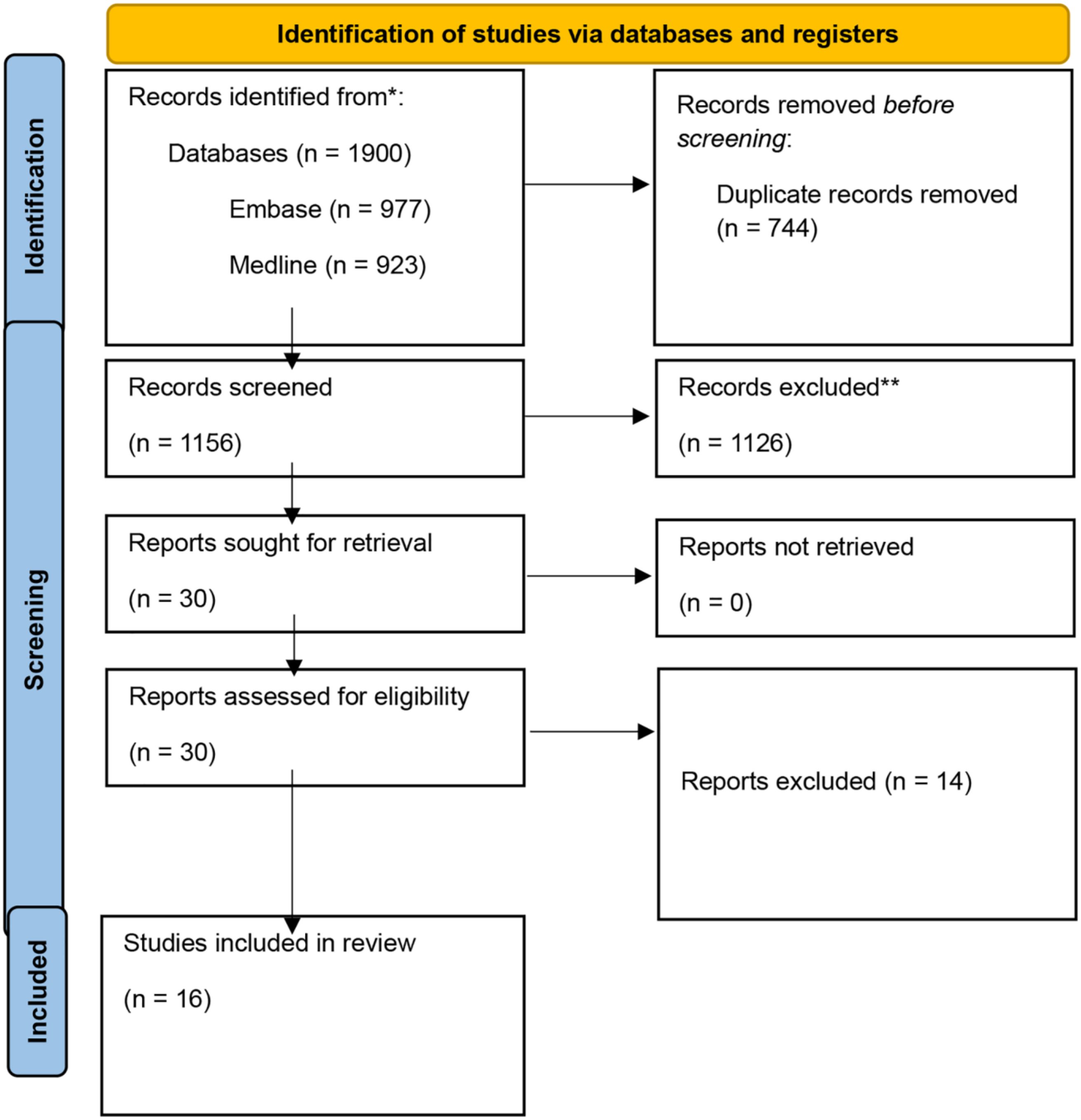

Supplementary Figure 1. PRISMA Flowchart.

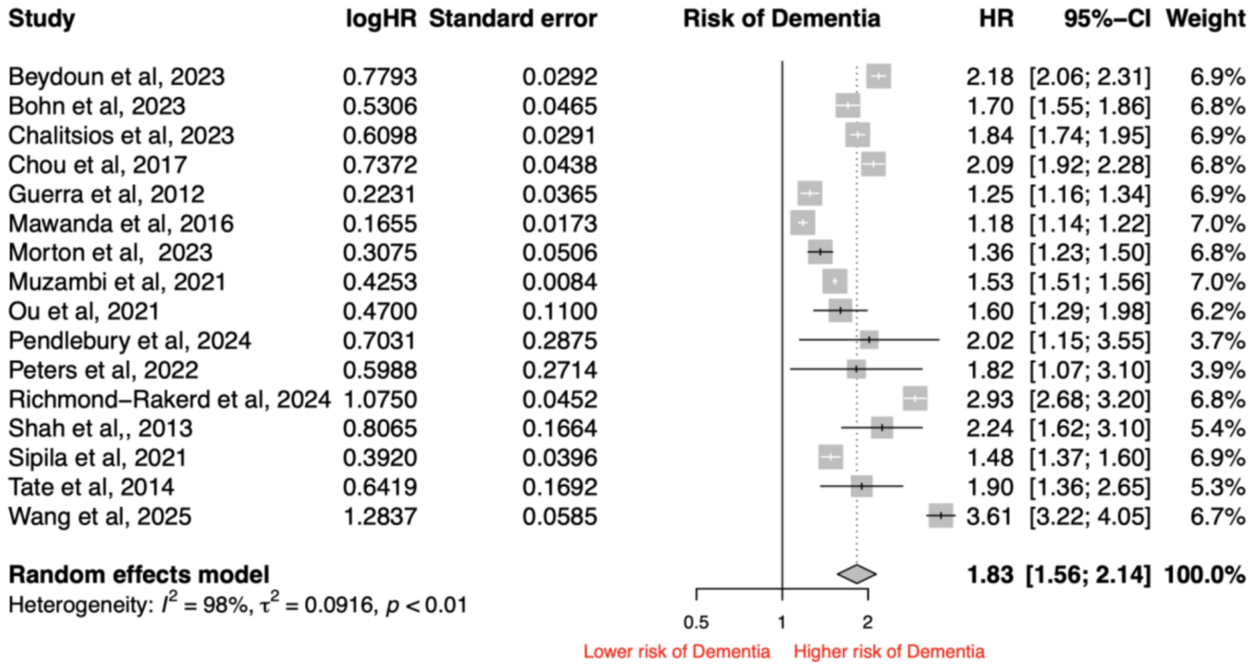

Supplementary Figure 2. Forest Plot of pooled Hazard Ratio (HR) for risk of all-cause Dementia in patients hospitalized with infections.

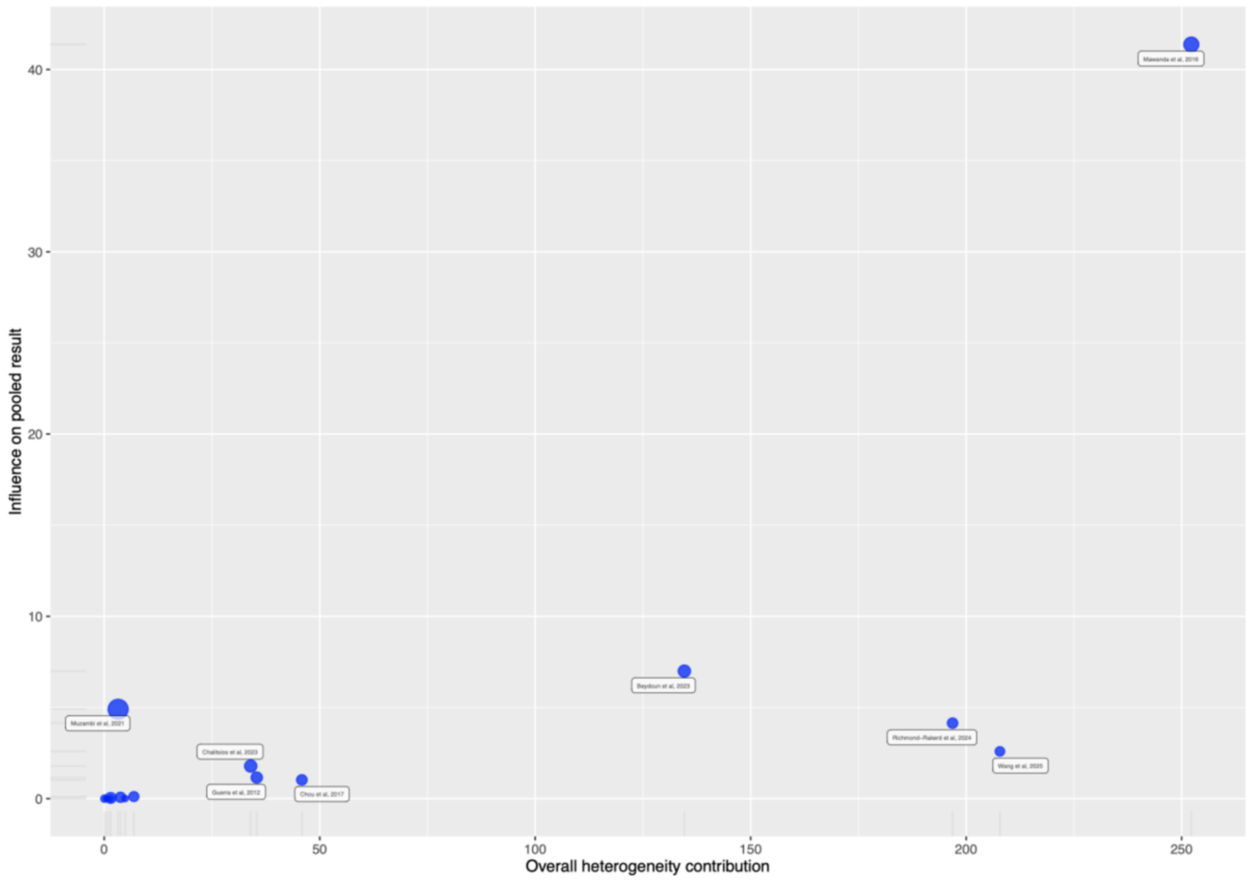

Supplementary Figure 3. Baujat Plot for hospitalization with infection and risk of all-cause Dementia based on pooled hazard ratios of 16 studies.

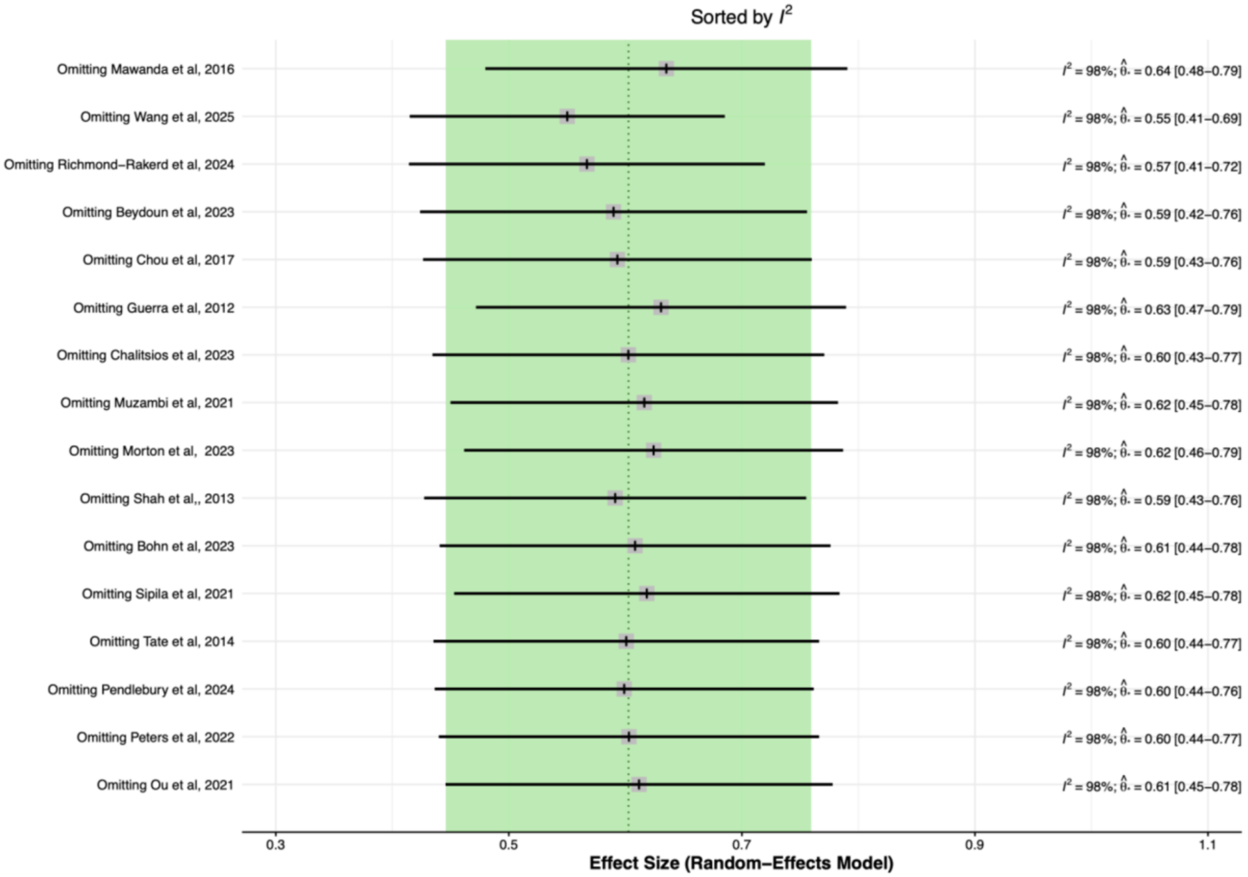

Supplementary Figure 4. Leave one out analysis for hospitalization with infection and risk of all-cause Dementia based on pooled hazard ratios of 16 studies. Effect size refers to treatment effect (logHR).

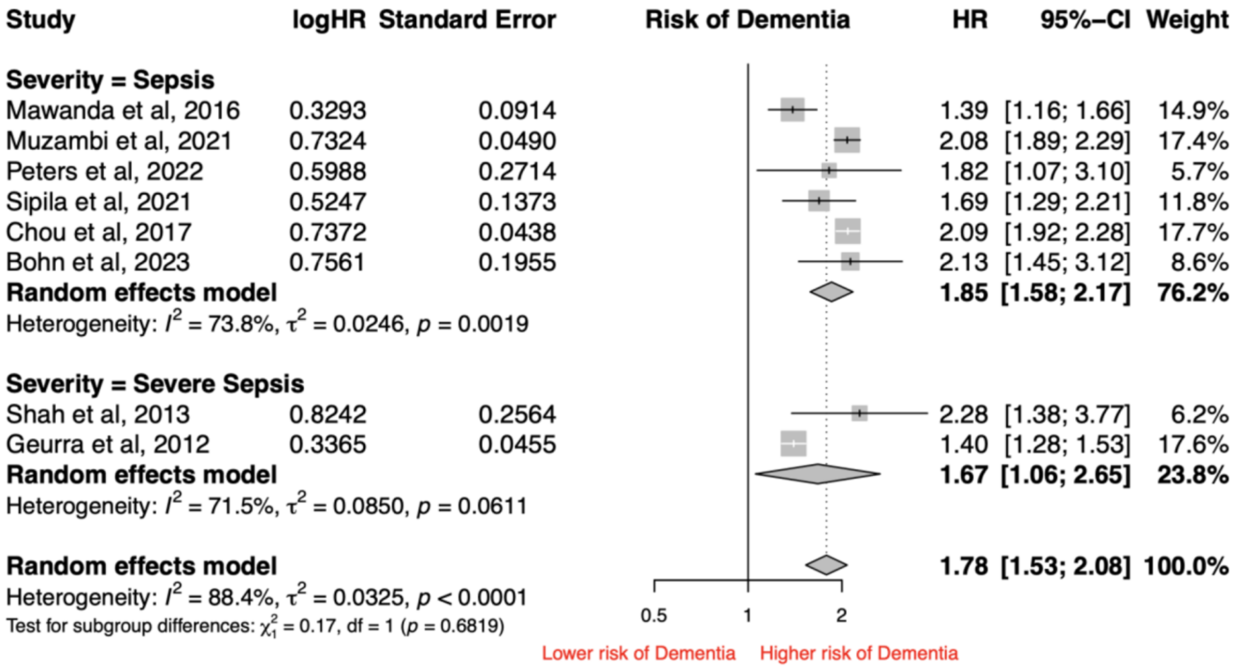

Supplementary Figure 5. Forest Plot of pooled Hazard Ratio (HR) for risk of all-cause Dementia in patients hospitalized with infections based on severity of sepsis.

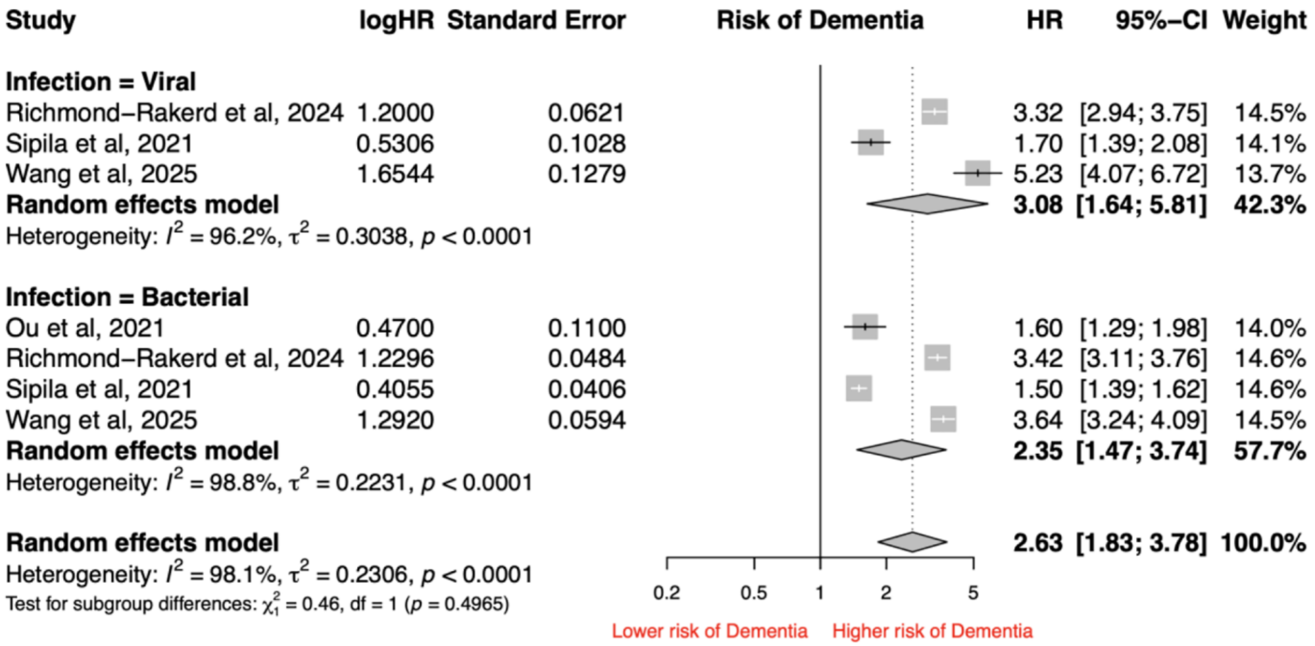

Supplementary Figure 6. Forest Plot of pooled Hazard Ratio (HR) for risk of all-cause Dementia in patients hospitalized with infections based on bacterial vs. viral infections.

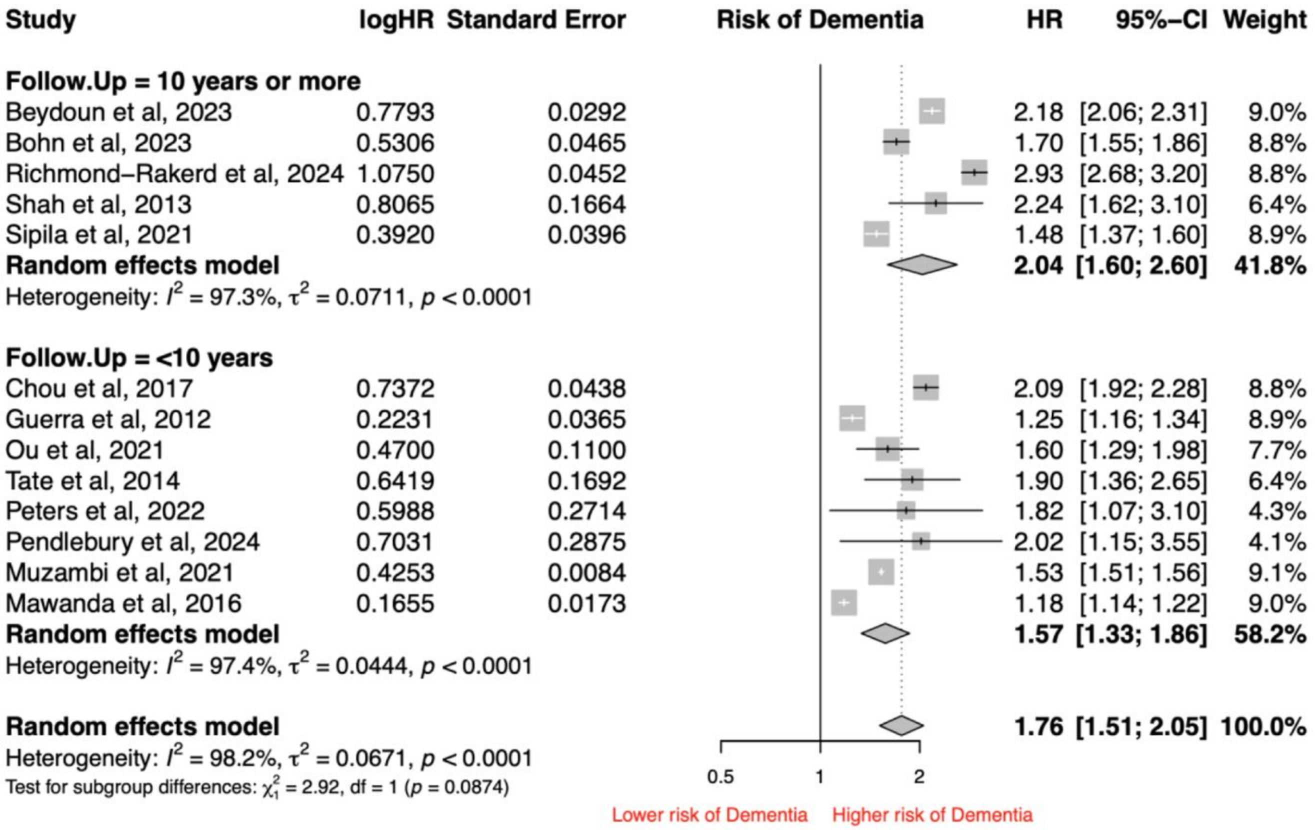

Supplementary Figure 7. Forest Plot of pooled Hazard Ratio (HR) for risk of all-cause Dementia in patients hospitalized with infections based on median duration of follow-up.

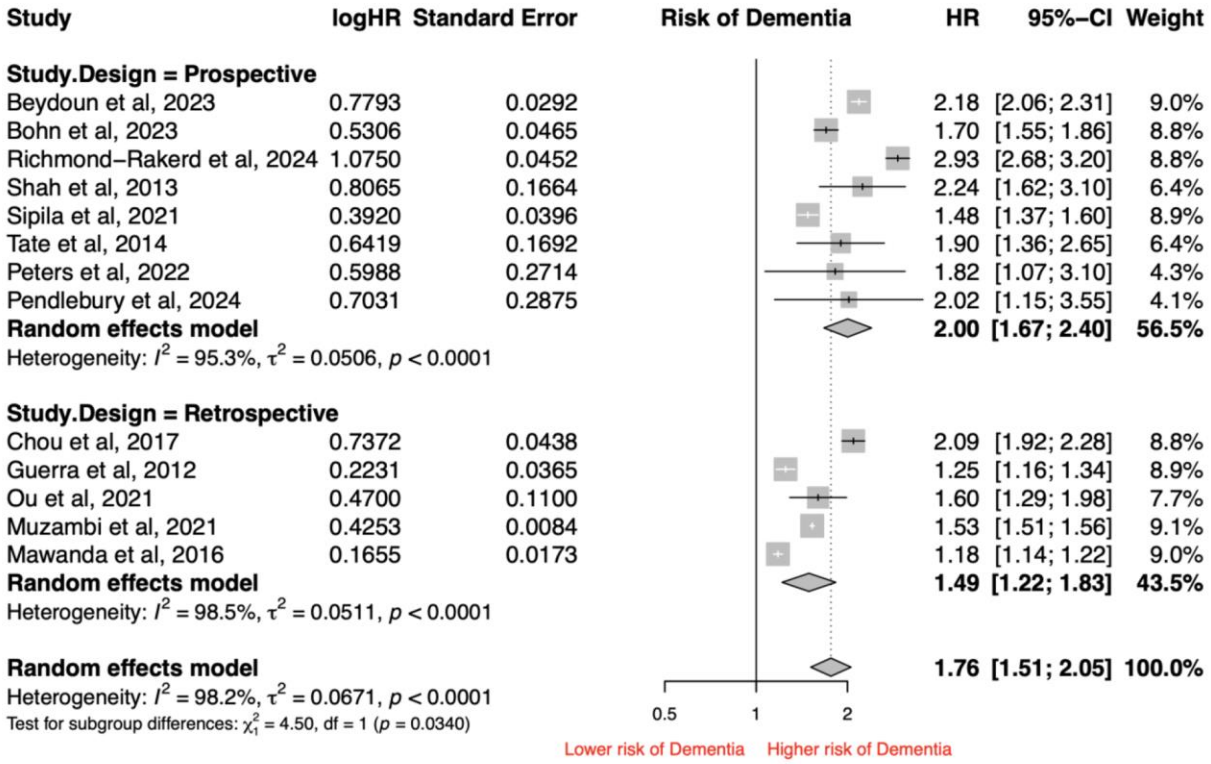

Supplementary Figure 8. Forest Plot of pooled Hazard Ratio (HR) for risk of all-cause Dementia in patients hospitalized with infections based on study design.

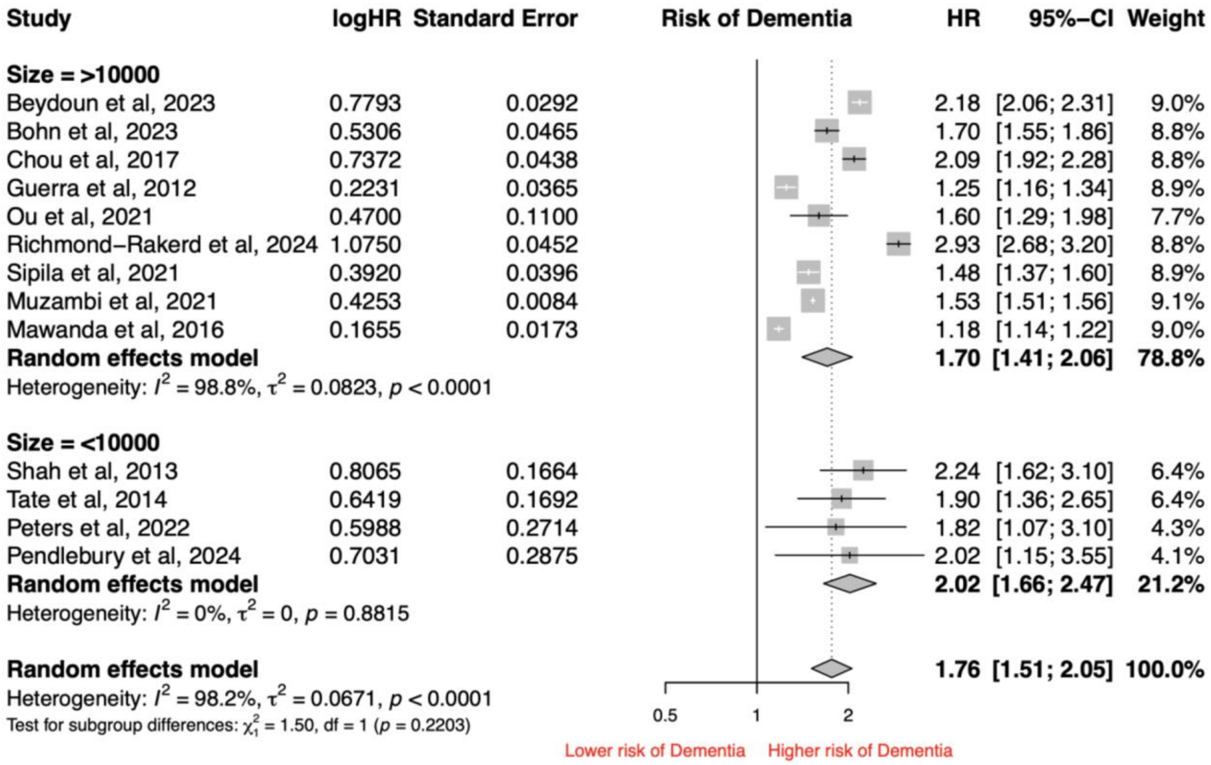

Supplementary Figure 9. Forest Plot of pooled Hazard Ratio (HR) for risk of all-cause Dementia in patients hospitalized with infections based on total sample size of study.

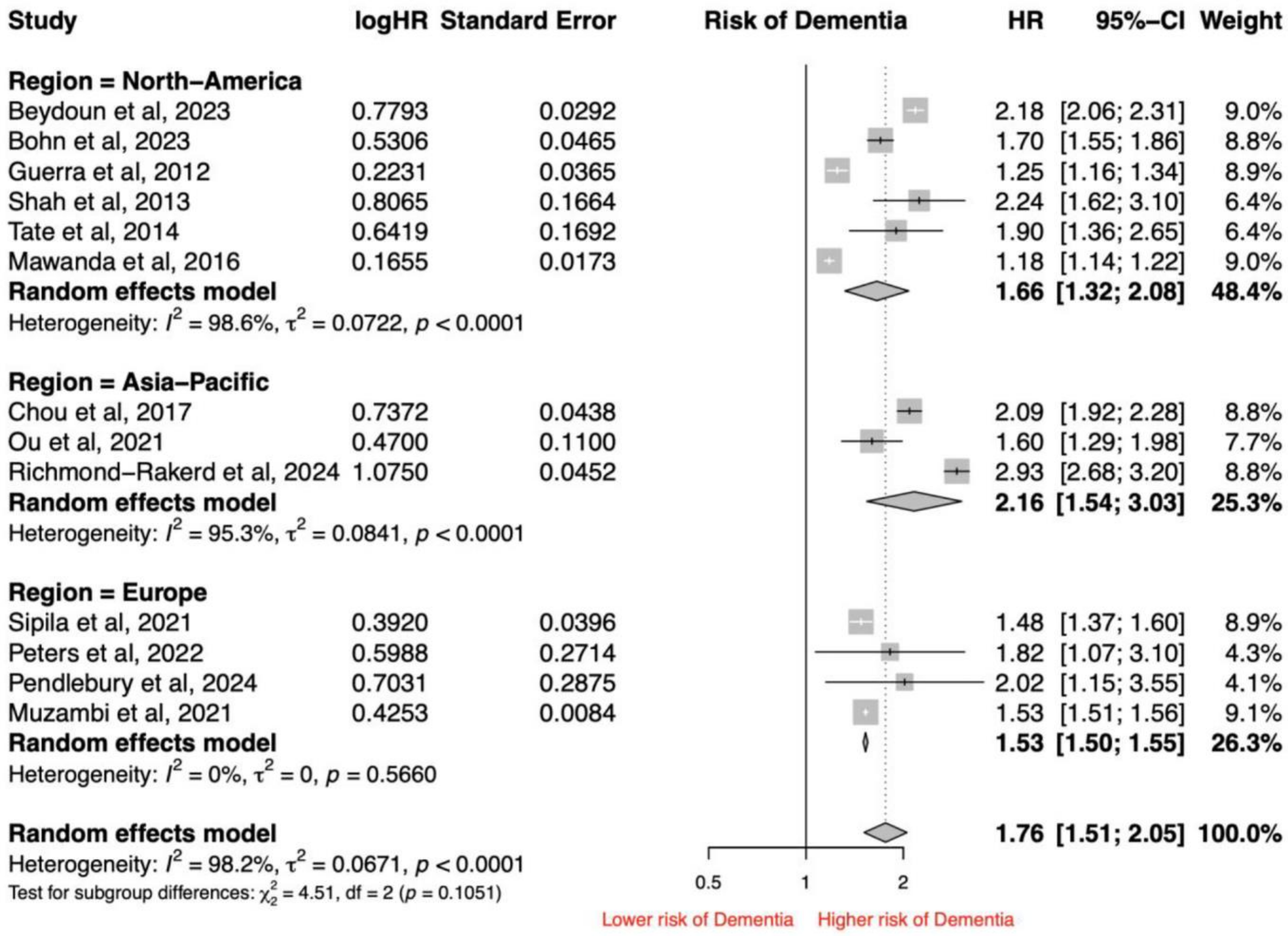

Supplementary Figure 10. Forest Plot of pooled Hazard Ratio (HR) for risk of all-cause Dementia in patients hospitalized with infections based on region of study conducted.
