# Supplementary material for: Hospitalization with infections and risk of Dementia: a systematic review and meta-analysis
Source: Aging (Albany NY). 2025 Oct 13;17(10):2561–81. doi: 10.18632/aging.206329 (PMC12606967; doi:10.18632/aging.206329)
Supplement: Supplementary Tables [file aging-17-10-206329-s002.pdf]

SUPPLEMENTARY TABLES

Supplementary Table 1. Search strategy.

EMBASE

31 March 2025

No.	Search terms	Hits
1.	‘infectio*':ab,ti AND ‘hospital*':ab,ti	431,070
2.	‘alzheimer disease*':ab,ti OR ‘dementia*':ab,ti	239,160
3.	#1 AND #2	1,979
4.	'case study*':ti OR 'systematic review*':ti OR 'meta analysis*':ti OR 'case report*':ti OR 'conference abstract'/it	6,278,846
5.	#3 NOT #4	977

MEDLINE

31 March 2025

No.	Search terms	Hits
1.	infectio*.ab,ti. AND hospital*.ab,ti.	259,720
2.	Alzheimer disease*.ab,ti. OR dementia*.ab,ti.	169,256
3.	1 AND 2	963
4.	Case study*.ti. or systematic review*.ti. or meta analysis*.ti. or case report*.ti.	814,436
5.	3 NOT 4	923

Breakdown

Total	1900
Duplicates	744
Remaining	1156

Supplementary Table 2. Study definitions of Hospitalization with infection, controls, Dementia and exclusion criteria.

Study	Hospitalisation with infection	Controls	Dementia	Exclusion criteria
Beydoun et al., 2023	Hospital admission records with primary or secondary infection diagnosis; ICD-10 codes used.	No hospital-treated infection	ICD-10 codes; algorithmically derived from medical records	Prevalent dementia or dementia that occurred prior to baseline assessment at baseline
Bohn et al., 2023	Hospitalizations were identified through telephone calls, local hospitals surveillance, and death interviews with proxies. Infection was coded by ICD-9/ICD-10.	Participants unexposed to hospitalisation with infection.	Identified via ICD-10 records, death certificates, in-person cognitive tests, and telephone interviews.	Dementia diagnosis at baseline
Chalitsios et al., 2023	ICD-10 codes J12–J18; only community-acquired pneumonia.	Matched non-pneumonia individuals	CPRD diagnosis codes for dementia; verified by expert.	Excluded prior cognitive impairment or dementia diagnosis. Hospital acquired pneumonia.
Chou et al., 2017	Diagnosis of septicaemia (ICD-9) in hospital records.	Matched individuals without septicemia	Insurance claim codes; includes AD and NAD.	Prior dementia diagnosis
Guerra et al., 2012	Diagnosis of infection and severe sepsis in medical records (ICD-9).	ICU survivors ≥66 years with no infection	Diagnosis of dementia recorded from fee-for-service insurance claims. ICD-9 dementia codes.	Patients with any diagnosis of dementia, mild cognitive impairment or general symptom mental loss. Patients discharged to hospice care and patients who died in the same quarter (three months) of their discharge date.
Mawanda et al., 2016	ICD-9 coded extra-CNS bacterial infections from hospital records.	Patients without extra-CNS bacterial infection diagnosis.	ICD-9 codes from inpatient and outpatient records.	Excluded patients with prior dementia or died during the baseline observation period. Patients with an ICD-9 code for other neurodegenerative disorders, cancer, chronic inflammatory diseases, or conditions associated with potentially reversible or non-neurodegenerative cognitive impairments.
Morton et al., 2023	GP-recorded and ICD-10 coded hospital infections	Stroke survivors without infections	Read and ICD-10 codes from CPRD and HES respectively	Excluded pre-stroke dementia and dementia within 3 months post-stroke.
Muzambi et al., 2021	ICD-10 codes, Read codes for infections and antibiotics prescription	≥65 years old with no infections	Read and ICD-10 codes from CPRD and HES respectively	Excluded those with prior dementia and cognitive impairment
Ou et al., 2021	Chlamydia pneumoniae infection using ICD-9 code from hospital records.	Matched individuals without Chlamydia pneumoniae infection	Alzheimer's Disease diagnosis based on ICD-9 codes from medical records.	Date of the AD diagnosis less than 1 year after the Chlamydia pneumonia diagnosis.
Pendlebury et al., 2024	ICD-10 codes for infections	No hospitalisation for infections	Diagnosed via clinical assessment, cognitive tests, hospitalisation documentation, death certificates. All dementia diagnoses were made using the DSM-IV criteria by a senior study physician with expertise in dementia.	Patients with major stroke or pre-existing dementia excluded.

Peters et al., 2022	Clinical diagnoses for infections derived from medical records of hospitals and general practitioners.	No hospital-treated infections.	DSM-IV criteria or reviewing all medical records and cognitive assessments.	Patient with dementia, nonvascular neurologic diseases, a life expectancy less than 6 months, and all conditions that hinder cognitive testing or neuroimaging.
Richmond-Rakerd et al., 2024	Diagnoses of infections using ICD-9 and ICD-10 codes from public inpatient hospital records.	No infection record across 30 years.	Hospital, pharmacy and death records via ICD-9 and ICD-10 codes	Patients with prior dementia or dementia within 1 month of infection diagnosis
Shah et al., 2013	ICD-9 codes for pneumonia	No hospitalisation for pneumonia	Neuropsychiatric testing, magnetic resonance imaging evaluation and physician assessment.	Excluded patients with prior dementia
Sipilä et al., 2021	ICD-10 codes from inpatient hospital discharge information for infections	No hospitalisation for infections	ICD-10 codes from hospital inpatient records, reimbursement for treatment of dementia, hospital outpatient records and death records	Excluded patients with prior dementia.
Tate et al., 2014	ICD-9 codes for pneumonia	No hospitalisation for pneumonia	Expert panel adjudication using the DSM-IV and NINCDS-ADRDA criteria for dementia.	Excluded patients with dementia, Parkinson's, cognitive enhancers or cholinesterase inhibitors use
Wang et al., 2025	ICD-10 codes extracted from the UK Biobank	No hospital-treated infection	ICD-10 codes from hospital admission records and death registries	Excluded patients with a history of neurodegenerative disease and missing covariate data

Abbreviations: ICD: International Classification of Diseases; CPRD: Clinical Practice Research Datalink; HES: Hospital Episode Statistics; DSM-IV: Diagnostic and Statistical Manual of Mental Disorders, Fourth Edition; NINCDS-ADRDA: National Institute of Neurological and Communication Disorders and Stroke and Alzheimer's Disease and Related Disorders Association.
